# Uterine Tonus Assessment by Midwives versus Patient self-assessment in the active management of the third stage of labor (UTAMP): study protocol for a randomized controlled trial

**DOI:** 10.1186/s13063-015-1111-5

**Published:** 2015-12-18

**Authors:** Joyce L. Browne, Nelson K. R. Damale, Tessa M. Raams, Eva L. Van der Linden, Ernest T. Maya, Roseline Doe, Marcus J. Rijken, Richard Adanu, Diederick E. Grobbee, Arie Franx, Kerstin Klipstein-Grobusch

**Affiliations:** Julius Global Health, Julius Center for Health Sciences and Primary Care, University Medical Center Utrecht, Heidelberglaan 100, 3584 CX Utrecht, The Netherlands; School of Medicine and Dentistry, College of Health Sciences, University of Ghana, Accra, Ghana; Department of Obstetrics and Gynecology, Korle Bu Teaching Hospital, Accra, Ghana; School of Public Health, College of Health Sciences, University of Ghana, Accra, Ghana; WHO Country Office Ghana, World Health Organization, Accra, Ghana; Department of Obstetrics and Gynecology, University Medical Center Utrecht, Utrecht, The Netherlands; Division of Epidemiology and Biostatistics, School of Public Health, Faculty of Health Sciences, University of the Witwatersrand, Johannesburg, South Africa

**Keywords:** Active management of third stage of labor, Uterine tonus assessment, Postpartum hemorrhage, Low and middle-income countries, Task shifting

## Abstract

**Background:**

Postpartum hemorrhage (PPH) is the leading cause of maternal mortality worldwide and accounts for one third of maternal deaths in low-income and middle-income countries. PPH can be prevented by active management of the third stage of labor (AMTSL), a series of steps recommended by the World Health Organization to be performed by skilled birth attendants (SBAs). Task shifting in the AMTSL step of uterotonic drugs administration to community health workers, traditional birth attendants and self-administration has been investigated as a strategy to increase access to quality obstetric care considering persistent SBA and facility-based delivery shortages. The aim of this study is to assess task shifting in the final step of AMTSL and compare uterine tonus assessment by a SBA to self-assessment.

**Methods and Design:**

The study is an individual-level two-arm non-inferiority randomized controlled trial (RCT). A total of 800 women will be recruited in Korle Bu Teaching Hospital in Accra, Ghana. Adult women in labor at term with an expected vaginal delivery who received antenatal instructions for self-assessment of uterine tonus will be eligible for inclusion. Women with an increased risk for PPH will be excluded. Women will be randomized to uterine tone assessment by a skilled birth attendant (midwife) or uterine tone self-assessment (with the safety back-up of a midwife present in case of PPH or uterine atony). Postpartum blood loss will be measured through weighing of disposable mats. The main study endpoints are PPH (≥500 ml blood loss), severe PPH (≥1000 ml blood loss), mean blood loss, and routine maternal and neonatal outcomes. Participants and caregivers will not be blinded given the nature of the intervention.

**Discussion:**

A reduction of PPH-related maternal mortality requires full implementation of AMTSL. Task shifting of uterine tone assessment may contribute to increased AMTSL implementation in (clinical) settings where SBAs capacity is constrained.

**Trial registration:**

Clinicaltrials.gov: NCT02223806, registration August 2014. PACTR: PACTR201402000736158, registration July 2014. University of Ghana, Medical School Ethical and Protocol Review Committee: MS-Et/M.8–P4.1/2014-2015

**Electronic supplementary material:**

The online version of this article (doi:10.1186/s13063-015-1111-5) contains supplementary material, which is available to authorized users.

## Background

### Postpartum hemorrhage is the leading cause of maternal death

Each year, approximately 287,000 women die because of preventable causes related to pregnancy and childbirth [1]. Almost all (99 %) of these deaths occur in low-income and middle-income countries (LMIC), with the largest burden in sub-Saharan Africa and South Asia [1].

The most common cause of maternal death is postpartum hemorrhage (PPH), defined as blood loss equal to or greater than 500 ml within 24 h after birth of the infant. PPH occurs in around 10.5 % of all deliveries and is responsible for more than 130,000 deaths annually [2, 3]. In sub-Saharan Africa, 33.9 % of maternal deaths are due to PPH [4].

The maternal mortality ratio of Ghana in 2013 was 380 maternal deaths per 100,000 live births [5]. The leading cause of maternal deaths was obstetric hemorrhage, with estimates ranging between 27 and 32 % [6–10].

Causes of PPH include uterine atony, uterine rupture, incisions, lacerations and coagulopathy, with uterine atony being the most common cause of PPH. Risk factors for atony include multiparity (five or more deliveries), polyhydramnios, fetal macrosomia, abnormal placentation, placental abruption, induced or prolonged labor and retained placental tissue [2, 11].

### AMTSL can prevent PPH

Whether a woman dies from PPH depends largely on access to timely and competent obstetric care [12]. PPH mostly occurs during the third and fourth stages of labor [2, 13], and active management of the third stage of labor (AMTSL) can prevent its occurrence. AMTSL is recommended as a series of steps performed by skilled birth attendants (SBAs) by the World Health Organization (WHO) and the international professional organizations of gynecologists and midwives [2, 13, 14]. The steps include the provision of uterotonic drugs (oxytocin or misoprostol) immediately upon fetal delivery, controlled cord traction, massage of the fundus of the uterus immediately after placental delivery in the absence of uterotonics, and routine assessment of the uterine tonus every 15 min for the first 2 h postpartum.

### Task shifting in AMTSL

AMTSL when performed by SBAs is the “gold standard” for prevention of PPH [2, 15] and can reduce excessive blood loss by 50–70 % [16]. This provides a challenge in many LMIC where the majority of women live in rural areas and deliver at home without SBAs available, often assisted by traditional birth attendants or family members who are not educated in obstetric care [17]. Task shifting of AMTSL to community health workers, traditional birth attendants, or self-administration has been explored as a strategy to increase access to quality obstetric care in response to the obstacles [18].

Of all AMTSL interventions, uterotonic drugs are the most effective in preventing PPH, although the relative contribution of each of the AMTSL components has not been well-studied [2, 19]. Oxytocin is the preferred uterotonic drug. However, as oxytocin is thermo-unstable, requires refrigeration and should be injected by SBAs, misoprostol is considered a safe, cheap and only slightly less effective alternative [20–23]. The WHO recommends the provision of misoprostol by community health workers for PPH prevention in rural areas and homebirths in absence of SBAs [2, 13]. The community distribution of misoprostol for self-administration by women is also widely explored as an option, but currently not recommended [24–26]. Controlled cord traction is contraindicated without assistance of a SBA [2]. To the best of our knowledge, there is currently no research or recommendation about the relative contribution of uterine tonus assessment for the prevention of PPH available, or the effect of task shifting of uterine tonus assessment [2, 13, 27]. Therefore, the aim of this non-inferiority randomized controlled trial (RCT) is to determine whether there is a difference in outcomes following routine uterine tone assessment every 15 min in the first 2 h postpartum performed by a SBA (midwife) compared to self-assessment by a patient.

## Methods/Design

### Study design

This study will be a pragmatic non-inferiority individual-level two-arm RCT comparing two strategies of AMTSL.

### Participants

The population base for the current study is pregnant women admitted in labor at Korle Bu Teaching Hospital (KBTH). KBTH has approximately 10,600 deliveries every year, approximately 200 per week. AMTSL has been implemented at KBTH. As a result of midwife understaffing, the routine assessment of the uterine tone for the first 2 h postpartum has been delegated to patients who received adequate training during the antenatal care (ANC) visits and who receive a repeated instruction after delivery. These women are regularly monitored by midwives. Yet, whether this task shifting is indeed equally effective has not been formally evaluated.

#### Inclusion criteria

In order to be eligible to participate in this study, the following criteria must be met: be at least 18 years of age, in labor with an expected vaginal delivery of a term singleton gestation, and recipient antenatal instructions on postnatal uterine tonus self-assessment.

#### Exclusion criteria

The following criteria will result in exclusion from study participation: operative delivery, severe anemia (<8 g/dL) within 2 weeks of delivery, risk factors for PPH (history of previous PPH, palpable uterine myoma, antepartum hemorrhage, anticipated breech delivery), intra uterine fetal death.

### Intervention

Participants will be randomly assigned to one of the two arms:

1. uterine tonus assessment by patient, every 15 min for the first 2 h (current standard practice).
2. uterine tonus assessment by a SBA (midwife), every 15 min for the first 2 h (guideline recommended practice).

### Study objectives and hypothesis

#### Primary objective

To determine whether there is a difference in incidence of PPH (≥500 ml blood loss) following routine uterine tone assessment every 15 min in the first 2 h postpartum performed by a SBA (midwife) compared to self-assessment by a patient.

#### Secondary objective

To determine whether routine uterine tonus assessment every 15 min in the first 2 h postpartum when performed by a SBA (midwife) compared to self-assessed by a patient, results in differences in incidence of severe PPH (≥1000 ml blood loss), differences in mean blood loss, use of additional uterotonics, use of other procedures for management of PPH (surgery, manual placenta tissue removal), blood transfusion, maternal resuscitation and maternal death.

#### Other study endpoints and parameters

Neonatal outcomes will be assessed by: Apgar scores at 1 and 5 mins, neonatal resuscitation or perinatal death.

The following socio-demographic and obstetric characteristics will be collected: age, marital status, employment, education, number of previous pregnancies, number of previous deliveries (live births and still births), number of children, present pregnancy history, medical and surgical history.

#### Hypothesis

It is hypothesized that there will be no difference in outcomes for the SBA-assessed group compared to the self-assessment arm.

### Sample size calculation and justification

PPH has an incidence of approximately 11.1 % in the Ghanaian population [28]. The hypothesis of this non-inferiority RCT is that there is no difference between the two treatments. To exclude a difference of between 5 to 7.5 % with a 1-sided alpha co-efficient of 5 % at a power of 80 %, between 978 and 436 participants would have to be randomized [29]. Given resource restrictions, our sample size will be 800 women, and powered to exclude a difference of 5.53 %.

As the intervention and outcome assessment take place on the same day, we expect no loss to follow-up.

### Study procedures

#### Recruitment and enrollment

Enrollment will take place at the ANC clinic and on the antenatal wards of KBTH after informed consent has been provided. Information about the study will be available at the ANC clinic and provided during the ANC education/training for uterine tonus assessment to familiarize women with the study (see Additional file 1 for the Standardized Operating Procedure for the training). Patients will be screened for eligibility based on the previously stated inclusion and exclusion criteria by trained research assistants. All patients will receive antenatal education on uterine tonus self-assessment.

#### Randomization, treatment allocation, and treatment concealment

Randomization occurs at presentation at the labor ward in established labor. If at this stage enrolled patients present with one of the exclusion criteria, they will be excluded from the study.

Participants will be assigned a study identification code based on consecutive enrollment. After the identification number has been assigned to a participant, a matching sealed opaque envelope in which the arm allocation is concealed can be opened by the research assistant. Women will be assigned an allocation arm while in labor, but before the onset of birth to facilitate the understanding of the arm they were allocated – as at time of full dilation or start of pushing, the ability to absorb the new information may have been reduced compared to an earlier stage of labor. The randomization list with blocks ranging between 2–6 identifiers will begenerated by the Data Management Department of the University Medical Center Utrecht using SAS software (SAS Inc., Cary, NC, USA). Additional randomization codes can be created to replace women who were excluded from the study because of complications, including caesarian sections.

Because of the nature of the intervention patients, care providers and research assistants will not be blinded.

#### Blood loss estimation

Assessment of blood loss will be performed by trained midwives by weighing blood collected after delivery of the placenta using disposable mats (“INCO pads”) of 90 x 60 cm. Scales will be standardized and calibrated. Blood loss will be measured up to 2 h postpartum.

#### Other outcomes

All other maternal endpoints will be obtained from maternal record books, surgery record books, antenatal booklet and interviews.

#### Disposal of medical waste

Mats used in the study will be disposed according to the standard procedures of disposal of medical waste at KBTH.

#### Standard of care during study participation

Participants will receive the standard of care during their participation in the study, including all standard care at KBTH associated with the development, prevention or treatment of PPH. Women who report inadequate uterine contraction to their midwife, will receive the standard of care according to PPH protocols of KBTH.

#### Handling and storage of data and documents

Data will be handled confidentially and anonymously through assigning a consecutive numeric identification (ID) code to participants. A separate subject identification list will be used to link the participant ID code to the participant names.

The local investigator(s) have access to the source data. The key to the code will remain with the coordinating investigator. In accordance to Good Clinical Practice rules, the dataset will be stored for 15 years.

### Statistical analysis

Descriptive statistics for participant characteristics (age, marital status, employment, education, number of previous pregnancies, number of previous deliveries (live births and still births), number of children, present pregnancy history, medical and surgical history) will be presented as frequencies and percentages for dichotomous and categorical variables, and means and standard deviations for continuous variables will be presented. Differences between treatment groups will be assessed using Student’s *t* test for continuous outcome data and the chi square test for discrete outcome data, and reported significant with a *p* value of < 0.05.

Analyses for the first and second research objectives between intervention group and outcomes will be according to intention-to-treat. Primary and secondary dichotomous, categorical and continuous outcome variables will be descriptively analyzed in an approach similar to the participant characteristics analysis.

For the blood loss-related outcomes of interest (PPH, severe PPH and mean blood loss), linear and logistic regression analysis will be used to analyze the difference between the two arms. Participant characteristics with significant differences between the two groups despite randomization will be considered confounders in a multivariate logistic or linear regression analysis. The lower limit of the 90 % confidence interval on the difference between the groups in frequency of the primary outcome will be used to assess non-inferiority for these three variables. Observations with missing outcome data will not be considered in these analyses.

### Ethical considerations

The study will be conducted according to the principles of the Declaration of Helsinki and in accordance with the Dutch Law Medical Research Involving Human Subjects Act and Good Clinical Practice. The trial has been approved by the University of Ghana Medical School Ethical and Protocol Review Committee (MS-Et/M.8–P4.1/2014–2015). After information is provided in Twi or English, participants need to provide written informed consent (signature or thumb print) before enrollment.

To ensure participant’s rights are protected, an independent gynecologist will be available for counseling.

#### Benefits and risks assessment

There are no direct benefits or incentives for participants who participate in this study. As the intervention group receives a midwife assessment of uterine tonus, the benefit of participating in this study is that PPH may be detected slightly earlier than may otherwise be the case. As the study takes places at the labor ward, the control arm will receive the same quality of care and will be monitored by the midwives as part of routine care. Because most of the data will be collected from collection books, the time investment is minimized as much as possible.

All participating women are enrolled in the national health insurance scheme. No data safety monitoring board was installed for this study. Details about safety reporting are available upon request.

### Public disclosure and publication policy

This trial has been publicly registered with the Pan-African Clinical Trials Registry (PACTR, PACTR201402000736158) and Clinicaltrials.gov (NCT02223806). Scientific publications will be developed under the direction of the investigators and disclosed unreservedly in international scientific peer-reviewed journals. The full protocol is available upon request.

## Discussion

### Diagnosis of PPH

Despite the diagnosis of PPH being dependent on the measurement of blood loss the professional organizations of gynecologists/obstetricians and midwives [14] and WHO [2] do not have a recommended standardized method to measure blood loss. Because visual estimation has been established to be inaccurate, especially with larger amounts of blood loss, other more objective methods to diagnose PPH have been developed. These methods are direct measurement, gravimetry and photometry. With direct measurement, blood is directly collected in a pan or collector bag. With photometry, the amount of blood loss can be determined by a process to convert blood pigment to alkaline hematine and subsequently analyzed. This method is referred to as the “gold standard.” However, in low resource settings the availability of this photometric method will be low and the time needed to extract all blood from linen and pads will take too long to diagnose PPH. A method commonly used in clinical practice as an alternative is gravimetry in which standard-sized pads (e.g. “INCOpads,” 90 x 60 cm) soaked with blood are weighed after delivery of the placenta, and grams converted to milliliters of blood loss. Although this method is accurate in diagnosing PPH, it can overestimate the incidence of PPH, because it is difficult to discriminate blood from other fluids like urine or amniotic fluid [30]. In this study, the gravimetric method for diagnosing PPH will be applied.

We acknowledge that this study’s sample size and (clinical) setting may not allow for immediate generalizability to other settings. Instead, larger studies which include other clinical settings and non-clinical settings will be necessary to externally validate the findings before implementation. Future research could also include the relative contribution of tonus assessment to the prevention of PPH, the optimal training program and timing of training. The experiences of pregnant women and health providers in task shifting of tonus assessment could also be considered in future studies.

### Trial status

The study will take place from April 2014 until July 2015 at the labor ward of Korle Bu Teaching Hospital in Accra, Ghana. Enrollment commenced in April 2014.

## Additional file

Additional file 1:
**Script of antenatal clinic health talk.** (DOC 23 kb)

## Abbreviations

AMTSL: active management of the third stage of labor
ANC: antenatal care
CCMO: Dutch Central Committee on Research Involving Human Subjects
ID: identification
KBTH: Korle Bu Teaching Hospital
LMIC: low-income and middle-income countries
PACTR: Pan-African Clinical Trials Registry
PPH: postpartum hemorrhage
RCT: randomized controlled trial
SBA: skilled birth attendant
UTAMP: Uterine Tonus Assessment by Midwives versus Patient
WHO: World Health Organization
