# Supplementary material for: Uterine Tonus Assessment by Midwives versus Patient self-assessment in the active management of the third stage of labor (UTAMP): study protocol for a randomized controlled trial
Source: Trials. 2015 Dec 18;16:580. doi: 10.1186/s13063-015-1111-5 (PMC4684626; doi:10.1186/s13063-015-1111-5)
Supplement: Additional file 1: — Script of antenatal clinic health talk. (DOC 23 kb) [file 13063_2015_1111_MOESM1_ESM.doc]

Supplement 1: Script **Antenatal Clinic Health Talk**

***By: midwife antenatal clinic or public health officer***

Health talk on uterine tonus assessment and massage after delivery

*Blood loss after delivery*

After a woman delivers there is some blood loss naturally from the womb through the vaginal canal. Most of the time this is not excessive and doesn’t cause any harm.

However in 1 out of 10 women blood loss could be more than average and sometimes if excessive may affect the health of the mother negatively requiring replacement by blood transfusion.

*Minimizing blood loss*

There is a way to minimize this blood loss by the midwife giving an injection to the mother just before delivering the placenta. This firms up the womb (out of which the baby and placenta came).

If the womb firms up following this injection and remains firm the blood loss is usually minimal.

*Checking the womb*

To ensure that the womb remains firm after this injection the mother who has just delivered is shown by the midwives the firmed up womb in her lower tummy. She is also shown how to check that the womb remains firmed up. The mother checking every quarter of an hour for the next two hours that it remains firm would significantly help reduce the blood loss and avoid consequences of excessive blood loss.

If on checking the womb is not firm, the mother and or midwife would massage the womb to come back to its firmed up condition.

*Action if the womb is lax*

At any time that the mother feels too much blood is coming out of her or the womb is lax, she has to quickly inform the midwife so that this can be checked and minimized.

*Teaching in antenatal clinic*

There is a model that can help women recognize the firmed up womb in the clinic as they wait for the antenatal clinic. These would help reinforce in their memory how the firmed up uterus feels like well before the delivery of the baby.

*BENEFITS / Side effects of intervention*

There is no side effects of checking the womb. It will rather help minimize blood loss, avoid excessive blood loss and blood transfusion.
